# Mesenchymal Stem Cells Obtained from Synovial Fluid Mesenchymal Stem Cell-Derived Induced Pluripotent Stem Cells on a Matrigel Coating Exhibited Enhanced Proliferation and Differentiation Potential

**DOI:** 10.1371/journal.pone.0144226

**Published:** 2015-12-09

**Authors:** Yu-Liang Zheng, Yang-Peng Sun, Hong Zhang, Wen-Jing Liu, Rui Jiang, Wen-Yu Li, You-Hua Zheng, Zhi-Guang Zhang

**Affiliations:** 1 Guanghua School of Stomatology, Hospital of Stomatology, Sun Yat-sen University, Guangdong Provincial Key Laboratory of Stomatology, Guangzhou, Guangdong, P.R. China; 2 The First Affiliated Hospital, Sun Yat-sen University, Guangzhou, Guangdong, P.R. China; 3 Zhongshan School of Medicine, Sun Yat-sen University, Guangzhou, Guangdong, P.R. China; 4 Guangdong Second Traditional Chinese Medicine Hospital, Guangzhou, Guangdong, P.R. China; French Blood Institute, FRANCE

## Abstract

Induced pluripotent stem cell-derived mesenchymal stem cells (iPSC-MSCs) serve as a promising source for cell-based therapies in regenerative medicine. However, optimal methods for transforming iPSCs into MSCs and the characteristics of iPSC-MSCs obtained from different methods remain poorly understood. In this study, we developed a one-step method for obtaining iPSC-MSCs (CD146+STRO-1+ MSCs) from human synovial fluid MSC-derived induced iPSCs (SFMSC-iPSCs). CD146-STRO-1-SFMSCs were reprogrammed into iPSCs by transduction with lentivirus-mediated Sox2, Oct-3/4, klf4, and c-Myc. SFMSC-iPSCs were maintained with mTeSR1 medium in Matrigel-coated culture plates. Single dissociated cells were obtained by digesting the SFMSC-iPSCs with trypsin. The dissociated cells were then plated into Matrigel-coated culture plate with alpha minimum essential medium supplemented with 10% fetal bovine serum, 1× Glutamax, and the ROCK inhibitor Y-27632. Cells were then passaged in standard cell culture plates with alpha minimum essential medium supplemented with 10% fetal bovine serum and 1× Glutamax. After passaging *in vitro*, the cells showed a homogenous spindle-shape similar to their ancestor cells (SFMSCs), but with more robust proliferative activity. Flow cytometric analysis revealed typical MSC surface markers, including expression of CD73, CD90, CD105, and CD44 and lack of CD45, CD34, CD11b, CD19, and HLA-DR. However, these cells were positive for CD146 and stro-1, which the ancestor cells were not. Moreover, the cells could also be induced to differentiate in osteogenic, chondrogenic, and adipogenic lineages *in vitro*. The differentiation potential was improved compared with the ancestor cells *in vitro*. The cells were not found to exhibit oncogenicity *in vivo*. Therefore, the method presented herein facilitated the generation of STRO-1+CD146+ MSCs from SFMSC-iPSCs exhibiting enhanced proliferation and differentiation potential.

## Introduction

Pluripotent stem cells, including embryonic stem cells (ESCs) and induced pluripotent stem cells (iPSCs), can maintain sufficient cell stores through unlimited self-renewal capacity and can differentiate into any cell type within the body. However, ethical controversy, immune rejection, and the potential oncogenicity of ESCs limit their applications in the clinical setting. Recently, the generation of iPSCs from adult somatic cells has allowed for the use of stem cells without the ethical dilemmas surrounding ESCs [1–3]. iPSCs have been shown to exhibit characteristics similar to ESCs in terms of morphology, surface markers expression, unlimited self-renewal, and differentiation potential [1–4]. Moreover, although iPSCs may be an inexhaustible cell source for regenerative medicine, they still carry the risk of oncogenicity.

Recent studies have shown that MSCs possess immunomodulatory properties and exhibit immune tolerance during transplantation [5,6]. MSCs can also migrate to damaged tissues and promote tissue repair by secretion of cytokines, chemokines, and extracellular matrix proteins [7]. Additionally, no reports have demonstrated the oncogenic characteristics of MSCs. Therefore, MSCs may be an attractive source for stem cell-based therapies in regenerative medicine. MSCs are present in diverse tissues, such as bone marrow, adipose tissue, dental pulp, synovium, synovial fluid, and muscle [8–13]. However, harvesting MSCs requires suitable donors and may be invasive. In addition, the number of MSCs acquired during harvest can be limited, and the capacity of MSCs for proliferation and differentiation decreases during cultivation or aging [14,15]. Therefore, establishing a stable, robust source of MSCs is imperative for successful therapeutic applications.

Recent studies have reported that MSCs can be obtained from iPSCs (iPSC-MSCs) using different protocols, such as coculture with the desired cell type, growth factors, or differentiation through embryoid bodies (EBs), indicating that iPSC-MSCs may be an attractive source for stem cell-based therapies in regenerative medicine [14–27]. However, the characteristics of iPSC-MSCs vary depending on the specific ancestor cells and transformation protocols, and appropriate methods for transforming iPSCs into MSCs have not been developed [14,17–19,25,27].

Koyama et al. [9] reported that stromal cell antigen 1 (STRO-1)+CD146+ synovial fluid-derived MSCs (SFMSCs) exist in the synovial fluid of the temporomandibular joint (TMJ) in patients with temporomandibular disorders (TMD). They proposed that SFMSCs may be a promising candidate cell type for cell-based strategies for articular cartilage repair. However, the expression of STRO-1 and CD146 in MSCs is not stable and depends on the donor, culture conditions, and cell status [28–30]. Additionally, Simmons et al. [31] reported that STRO-1 antigen is progressively lost during culture and aging. Furthermore, the amount and status of SFMSCs obtained from patients with TMD shows great interpatient variability.

In this study, we reprogrammed SFMSCs that had lost STRO-1 and CD146 antigens during culture in vitro into iPSCs by reprogramming techniques with Sox2, Oct-3/4, klf4, and c-Myc. We then transformed these iPSCs into MSCs on Matrigel-coated plates.

## Materials and Methods

### Ethics statement

Individuals who donated synovial fluid provided written informed consent to participate in this study. The study protocol was approved by the Institutional Ethics Board of the Hospital of Stomatology, Sun Yat-sen University, Guangzhou, China.

### Measurement of cytokine levels in synovial fluid and culture of SFMSCs

Synovial fluid samples were collected from three TMD patients who did not respond to conservative treatment during arthrocentesis of the TMJ. These patients had no other systemic diseases, and their ages ranged from 21–58 years old. One patient was a man, and two patients were women. The collection and culture procedure was similar to that in our previous study [32]. Briefly, the upper joint compartment was expanded with 2.0 mL of lidocaine containing 25% vitamin B12 using a #8 needle and syringe for local anesthesia, and the fluid was then withdrawn. The collected synovial fluid samples were centrifuged at 300 × *g* for 5 min. After centrifugation, the cells were plated on culture plates with complete culture medium (alpha minimum essential medium supplemented with 10% fetal bovine serum [Gibco, USA] and 1× Glutamax [Gibco]) and incubated at 37°C in 5% CO2. After 48 h, the medium was withdrawn to remove non-adherent cells and replaced with fresh medium. Cells were then grown for about 2 weeks, after which the cells were passaged every 7 days at a density of 500 cells/cm^2^. The supernatants were used for cytokine level detection using a method similar to that described in previous studies [33,34]. Briefly, the supernatants were centrifuged (4°C, 10 min, 3000 × *g*) before measurement. The optical density (OD) of the solution before injection and after aspiration was measured at 550 nm using a microplate reader (Infinite200; Tecan). 50 μL of each supernatant was used to detected the cytokine level (IL-8, IL-1β, IL-6, IL-10, TNF, IL-12p70) with a Cytometric Bead Array kit (BD; cat. no.: 551811) according to the manufacturer’s instructions. The concentration of each cytokine in synovial fluid was calculated using the following formula: C_final_ = C_after_ / ([OD_before_−OD_after_] / OD_before_), where C_final_ indicates the synovial fluid concentration, C_after_ indicates the aspirate solution concentration, OD_after_ indicates the absorbance of solution after aspirate, and OD_before_ indicates the absorbance of solution before injection.

### Generation and culture of SFMSC-iPSCs

The expression vectors (pMXs-Oct3/4, pMXs-SOX2, pMXs-KLF4, and pMXs-c-MYC), packaging vector (pUMVC), and envelop vector (pVSV-G) were a gift from Prof. Qi Zhang of The Third Affiliated Hospital, Sun Yat-sen University. The protocol for SFMSC-iPSC induction was similar to that described in a previous report [1]. Briefly, 293FT cells (Invitrogen) were plated at 4 × 10^6^ cells per 100-mm dish with 9 mL high-glucose Dulbecco’s modified Eagle medium supplemented with 10% fetal bovine serum (HG-DMEM-FSB) and incubated overnight. The culture medium was replaced 2 h before transfection. Cells were transfected with pMXs, pUMVC, and pVSV-G using the calcium phosphate precipitation method. The culture medium was then replaced at 12 h after transfection. Additionally, 48 and 72 h after transfection, the supernatant was collected and filtered through a 0.45-μm pore-size cellulose acetate filter (Millipore). SFMSCs (passage 6) from the three TMD patients were plated at 5 × 10^4^ per well in a six-well plate with 2 mL HG-DMEM-FBS on the third day after transfection. The culture medium was then replaced after another 24 h with the virus-containing supernatant (collected after 48 h) and supplemented with 8 mg/mL polybrene. Cells were incubated for 12 h, and culture medium was replaced with 2 mL HG-DMEM-FBS. On the fifth day, the culture medium was replaced with the virus-containing supernatant (collected after 72 h) supplemented with 8 mg/mL polybrene. Twelve hours later, the culture medium was replaced with 2 mL HG-DMEM supplemented with 10% defined fetal bovine serum for ESCs (Gibco; HG-DMEM-DFBS). Ten days after transfection, SFMSCs were harvested by trypsinization and replated at 1 × 10^4^ cells per 100-mm dish on a feeder layer of mouse embryonic fibroblasts (MEFs) with HG-DMEM-DFBS supplemented with 50 μg/mL vitamin C and 1 mM valproic acid. The medium was changed every day. Twenty days after transfection, colonies were picked up and transferred into 1 mL of mTeSR1 medium for human iPSCs and ESCs (Stem Cell, USA). The cell suspension was then transferred into 6-well plates coated with Matrigel (HESC-QUALIFIED, BD) with 2 mL mTeSR1 medium per well. One colony was chosen randomly from each patient was further analyzed in this study.

### Generation and culture of SFMSC-iPSC-MSCs

SFMSC-iPSCs were harvested using 0.05% trypsin (Gibco). The dissociated cells were then seeded at a density of 1 × 10^4^ cells/cm^2^ in cell culture plates coated with Matrigel (1:500, HESC-QUALIFIED, BD; Corning) with complete culture medium supplemented with the ROCK inhibitor Y27632 (10 μM). The culture medium was replaced every 3 days. Adherent cells were then harvested using 0.05% trypsin and seeded in standard cell culture plates with complete culture medium after 1 week at a density of 500 cells/cm^2^. We defined these cells as SFMSC-iPSC-MSCs (passage 1).

### Cell proliferation and senescence assay

SFMSCs (passage 6) and SFMSC-iPSC-MSCs (passage 4) were seeded in triplicate at a density of 900 cells/cm^2^ in 12-well plates. The culture medium was changed every other day. Cell numbers were counted daily during the first week using a Z2 Coulter Particle count and size analyzer (Beckman Coulter). Repeated measurements (n = 3) were carried out for each well. To assess population doubling (PD) and calculate the doubling time (DT), cell number and culture times were measured and recorded. PD and DT were calculated using the following formula: PD = (lnN–lnN0) / ln2, DT = T / PD, where N is the cell number at the end point, N0 is the cell number at the initial time, and T is the time interval. A senescence β-Galactosidase Staining Kit (Beyotime Biotechnology, China) was used to detect the senescence of SFMSCs (passage 6) and SFMSC-iPSCs cultured in 12-well plates with complete culture medium according to the manufacturer’s instructions.

### Karyotype analysis

After passaging for 5 days, SFMSC-iPSC-MSCs were treated with colchicine at 37°C for 30 min, harvested with 0.25% trypsin, treated with 0.075 M KCl solution, and fixed in carnoy fluid. Fixed cells were analyzed by the G-band method.

### Flow cytometric analysis of cell surface antigens

SFMSCs (passage 6) and SFMSC-iPSC-MSCs (passage 4) were harvested using 0.25% trypsin. The phenotypes of both cells were studied by flow cytometry analysis for a panel of different surface markers: fluorescein isothiocyanate-conjugated anti-human CD90 (1:20; BD Biosciences), allophycocyanin-conjugated anti-human CD73 (1:11; Miltenyi Biotec), allophycocyanin-conjugated anti-human CD105 (1:20; BD Biosciences), phycoerythrin-conjugated anti-human CD44 (1:20; BD Biosciences), phycoerythrin-conjugated anti-human CD45/CD34 CD11b/CD19/HLA-DR (1:20; BD Biosciences), phycoerythrin-conjugated anti-human CD146 (1:5; BD Biosciences). Briefly, each sample was transferred to a 1.5-mL Eppendorf tube (about 5 × 10^5^ cells per tube), then incubated with the antibodies or negative control antibodies for 30 min at 4°C with a total volume of 100 mL per tube. After incubation with antibodies, the cells were washed with PBS and resuspended in 1 mL PBS. At least 10,000 events were acquired for each sample using an FC500 flow cytometer and MXP software (Beckman Coulter) according to the manufacturer’s instructions. Analysis of flow cytometry data was performed using CXP Software (Beckman Coulter).

### Alkaline phosphatase (AP) staining and immunofluorescence

AP staining was performed using an Alkaline Phosphatase kit (Millipore, USA). For immunofluorescence, cells were fixed with PBS containing 4% paraformaldehyde for 30 min at room temperature. After washing with PBS, the cells were treated with 0.1% Triton X-100 for 15 min at room temperature before blocking for 2 h with 1 mL of 1× PBS containing 5% bovine serum albumin (BSA). Primary antibodies included mouse anti-human NANOG (1:200, Novus), mouse anti-human OCT4 (1:200, Novus), rabbit anti-human SOX2 (1:50, Novus), mouse anti-human SSEA4 (1:500, Cell Signaling Technology, Beverly, MA, USA), mouse anti-human TRA-1-60 (1:50, Novus), mouse anti-human TRA-1-81 (1:50, Novus), rabbit anti-human VCAM-1 (1:100, Santa Cruz Biotechnology, Santa Cruz, CA, USA), mouse anti-human STRO-1 (1:50, Novus). The secondary antibodies used were DyLight 488-TFP ester-conjugated goat anti-rabbit IgG antibody (1:100, EarthOx), fluorescein isothiocyanate-conjugated rabbit anti-mouse IgM antibody (1:200, eBioscience), fluorescein isothiocyanate-conjugated goat anti-mouse IgG antibody (1:100, Santa Cruz Biotechnology). Nuclei were stained with 1 μg/mL DAPI (Cell Signaling).

### Differentiation assays *in vitro*

For formation of embryoid bodies (EBs), SFMSC-iPSCs were harvested by treating with collagenase IV. The clumps of cells were seeded in untreated 6-well plates (NEST) in mTSeR1 culture medium. The medium was changed every other day. After 8 days in floating culture, EBs were examined under an inverted phase-contrast microscope (Axiovert 40, Zeiss).

#### For osteogenic differentiation

SFMSCs and SFMSC-iPSC-MSCs were seeded at a density of 5000 cells/cm^2^ in 6-well plates and incubated in complete culture medium at 37°C in 5% CO_2_. After 24 h, the medium was replaced with osteogenic induction medium consisting of H-DMEM (Gibco), 10% FBS (Gibco), 10 mM sodium β-glycerophosphate (Santa Cruz Biotechnology), 10 nM 1,25-dihydroxyvitamin D3 (Sigma), and 50 μg/L ascorbic acid-2-phosphate (Wako, Japan). The medium was replaced every 3 days. After induction, medium was assessed by Alizarin Red staining by incubation with a fresh 0.1% Alizarin Red solution for 30 min at 37°C.

#### For adipogenic differentiation

SFMSCs and SFMSC-iPSC-MSCs were seeded at a density of 5000 cells/cm^2^ in 6-well plates and incubated in complete culture medium at 37°C in 5% CO_2_. After 24 h, the medium was replaced with adipogenic induction medium consisting of H-DMEM (Gibco) containing 10% FBS (Gibco), 200 mM indomethacin (Sigma-Aldrich, St. Louis, MO, USA), 0.5 mM isobutyl methylxanthin (MP Biomedicals), 1 mM dexamethasone (MP Biomedicals), and 10 mg/mL insulin (MP Biomedicals). The medium was replaced every 3 days. After induction, cell staining was performed with a fresh 0.5% sudan black B solution for 3 min.

#### For chondrogenic differentiation

Approximately 3 × 10^5^ SFMSCs and SFMSC-iPSC-MSCs were transferred to 15-mL centrifuge tubes and then centrifuged at 450 × *g* for 8 min. Then, 400 mL chondrocyte differentiation induction medium consisting of H-DMEM (Gibco), 1× ITS-A (Gibco), 100 nM dexamethasone (MP Biomedicals), 50 mM ascorbic acid (Sigma-Aldrich), 40 mg/mL proline (Sigma-Aldrich), and 10 ng/mL transforming growth factor-beta 1 (PeproTech) was added. The medium was refreshed every 3 days. Chondrogenic differentiation was assessed by histological staining. Paraffin-embedded cartilage nodules were sliced at 5 μm thickness. After deparaffinization and rehydration, the sections were stained with 0.1% Safranin O solution for 5 min. For glycosaminoglycan quantification assays, 3 × 10^5^ SFMSCs and SFMSC-iPSC-MSCs were transferred into 15-mL centrifuge tubes for chondrogenic differentiation. After culturing for 21 days, each cartilage nodule was digested with 100 μL proteinase K (50 μg/mL; Sigma) at 60°C overnight. Proteinase K was then inactivated by heating the solution for 10 min at 90°C, and the solution was then centrifuged (4°C, 30 min, 12000 × *g*). This preparation was used to quantify glycosaminoglycans as reported previously [35]. Briefly, working buffer was prepared by dissolving 16 mg DMMB in 25 mL ethanol, filtering through filter paper, adding 100 mL of a solution containing 1 M GuHCl, 1 g sodium formate, and 1 mL of 98% formic acid, and then adding distill water to a final volume of 500 mL. Next, 0.5 mL working buffer was added to 50 μL preparation, and the mixture was shaken and centrifuged (4°C, 10 min, 12000 × *g*). The supernatant was then discarded, and the pellet was dissolved with 500 μL decomplexation solution (4 M GuHCl solubilized in 50 mM sodium acetate containing 10% propanol solution buffer [pH 6.8]) and shaken for 30 min. Known amounts (1, 2, 4, 8, 10 μg/mL) of chondroitin sulfate (Sigma) were used as the standard solution. The OD of the solution was measured at 656 nm using a microplate reader (Infinite200; Tecan).

### Tumor-generation assay *in vivo*

SFMSC-iPSCs and SFMSC-iPSC-MSCs were harvested by collagenase IV and trypsin treatment, then collected into tubes, centrifuged, and resuspended in DMEM/F12. Cells were injected subcutaneously into the dorsal flanks of three SCID mice (China). Twelve weeks after injection, tumors were dissected and fixed with 4% paraformaldehyde. Paraffin-embedded tissue was sliced and stained with hematoxylin and eosin.

### Immunohistochemistry

Sections of cartilage nodule tissue were deparaffinized and rehydrated and then treated with 3% H_2_O_2_. Slides were next incubated in 0.01 M citrate buffer for 20 min at 94–98°C and blocked with 5% BSA. Primary antibodies included rabbit anti-human collagen type II antibodies (1:80, Sigma-Aldrich). The staining was visualized using a microscope (Axioskop 40, Zeiss) by applying streptavidin-biotin complex reagent (Boster) and 3,3′-diaminobenzidine (DAB; Boster) after the treatment with a solution of biotinylated goat anti-rabbit IgG (Boster). Sections were treated using the same process but without incubation with a primary antibody as the negative control.

### Gene expression analysis by real time-PCR

Total RNA was isolated using TRIzol reagent (Roche). cDNA was synthesized using a Transcriptor First Strand cDNA Synthesis Kit (Roche) according to the manufacturer’s instructions. Relative mRNA expression levels were determined by applying a SYBR Green qPCR kit (Roche) using GAPDH as the reference control. The primers for these genes are listed in Table 1. The 2^-ΔΔCT^ method was used to analyze the relative gene expression levels by normalizing with GAPDH as an endogenous control and calibrating according to the primer efficiency. ΔΔCT was calculated using the following formula: (Ct_target gene_−Ct_gapdh_)sample–(Ct_target gene_−Ct_gapdh_)control.

**Table 1:** Oligonucleotide primers used in qPCR.

Gene	Primer sequence
*GAPDH*	Forward: GACAGTCAGCCGCATCTTCT
	Reverse: TTAAAAGCAGCCCTGGTGAC
*RUNX-2*	Forward: TCAACGATCTGAGATTTGTGGG
	Reverse: GGGGAGGATTTGTGAAGACGG
*OCN*	Forward: CCACCGAGACACCATGAGAG
	Reverse: TCAGCCAACTCGTCACAGTC
*PPARG2*	Forward: GCAAACCCCTATTCCATGCTG
	Reverse: CACGGAGCTGATCCCAAAGT
*LPL*	Forward: CAAGAGTGAGTGAACAAC
	Reverse: AATTATGCTGAAGGACAAC
*SOX9*	ACACACAGCTCACTCGACCTTG
	AGGGAATTCTGGTTGGTCCTCT

**Abbreviations:** *GAPDH*, glyceraldehyde-3-phosphate dehydrogenase; *RUNX-2*, runt-related transcription factor 2; *OCN*, osteocalcin; *PPARG2*, peroxisome proliferator-activated receptor gamma, transcript variant 2; *LPL*, lipoprotein lipase; *SOX9*, sex determining region Y-box 9.

### Statistical analysis

Numerical data are expressed as means ± standard deviations (SDs). Statistical significance was evaluated via Student’s t-test, and *p*-values of less than 0.05 were considered significant.

## Results

### Patient information and *in vitro* characterization of SFMSCs

**Fig 1:**
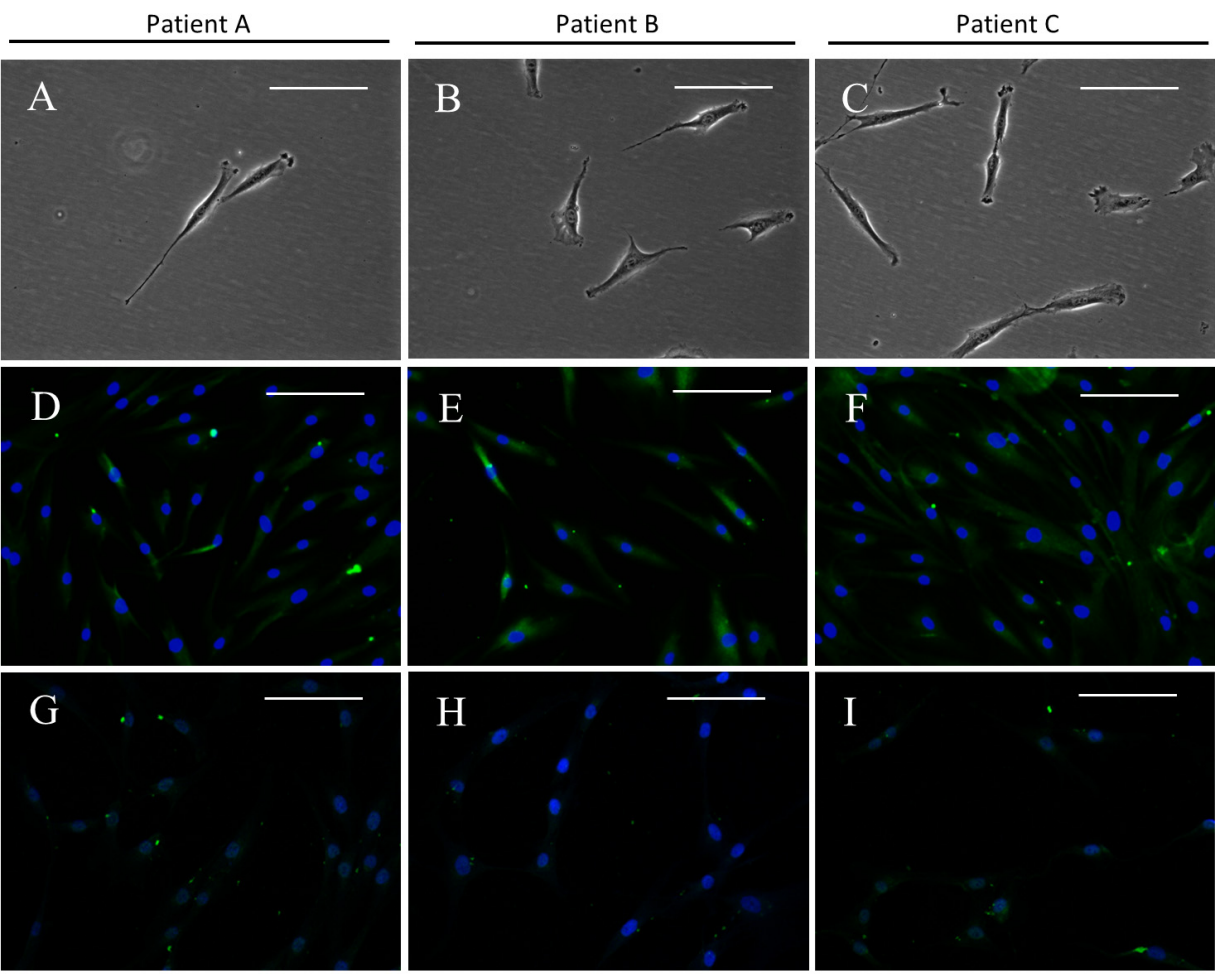
SFMSCs. (A–C) Microscopic image showing the typical morphology of SFMSCs. (D–F) Immunofluorescent staining of SFMSCs showing positive expression of STRO-1 at passage 2. (G–I) Immunofluorescent staining of SFMSCs showing decreased expression of STRO-1 at passage 6. Scale bars = 100 μm.

**Fig 2:**
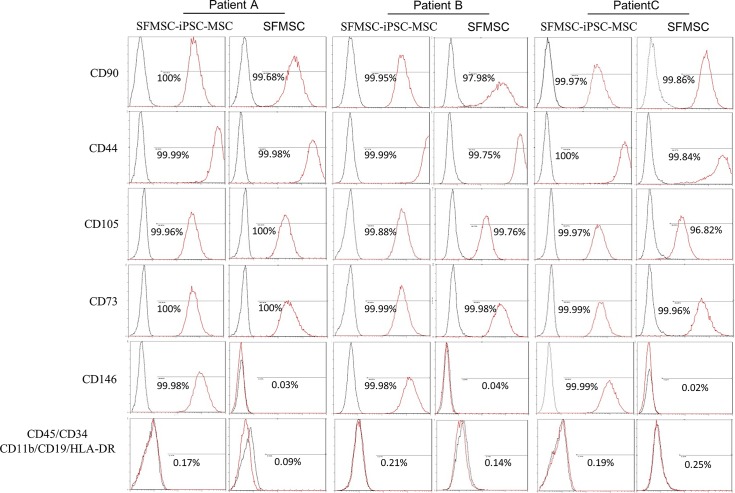
Flow cytometric analysis of SFMSCs and SFMSC-iPSC-MSCs. Both SFCs and SFMSCs expressed typical MSCs surface markers, including CD90, CD44, CD105, and CD73. CD45, CD34, CD11b, CD19, and HLA-DR were not detected on the surfaces of these cells. SFMSC-iPSC-MSCs expressed CD146. The black lines represent negative controls, and the red lines are the results for the experimental groups.

A summary of the patients’ characteristics is shown in Table 2. After culturing the diluted synovial fluid samples for a few days, SFMSC proliferation was observed in culture, and the cells exhibited a typical fibroblastic spindle shape (Fig 1A–1C). STRO-1 was detected in these SFMSCs at passage 2 (Fig 1D–1F) but was almost completely absent after ex vivo expansion at passage 6 (Fig 1G and 1I). Flow cytometric analysis showed that ex vivo-expanded SFMSCs (passage 6) expressed CD90, CD105, CD73, and CD44. CD146, CD45, CD34, CD11b, CD19, and HLA-DR were not detected on the cells (Fig 2).

**Table 2:** Summary of patients’ characteristics.

Patient no.	Age (years)(years)	Sex	Lateral	Diagnosis	Cytokine level (pg/mL)					
					IL-8	IL-1β	IL-6	IL-10	TNF	IL-12p70
A	21	F	Left	Anterior disk displacement without reduction	42.39	65.50	21.92	21.26	54.37	87.29
B	32	M	Right	Osteoarthrosis	282.24	424.01	270.81	215.54	321.55	482.77
C	58	F	Left	Osteoarthrosis	79.99	126.28	78.08	58.33	96.42	188.73

### Generation and characterization of SFMSC-iPSCs

**Fig 3:**
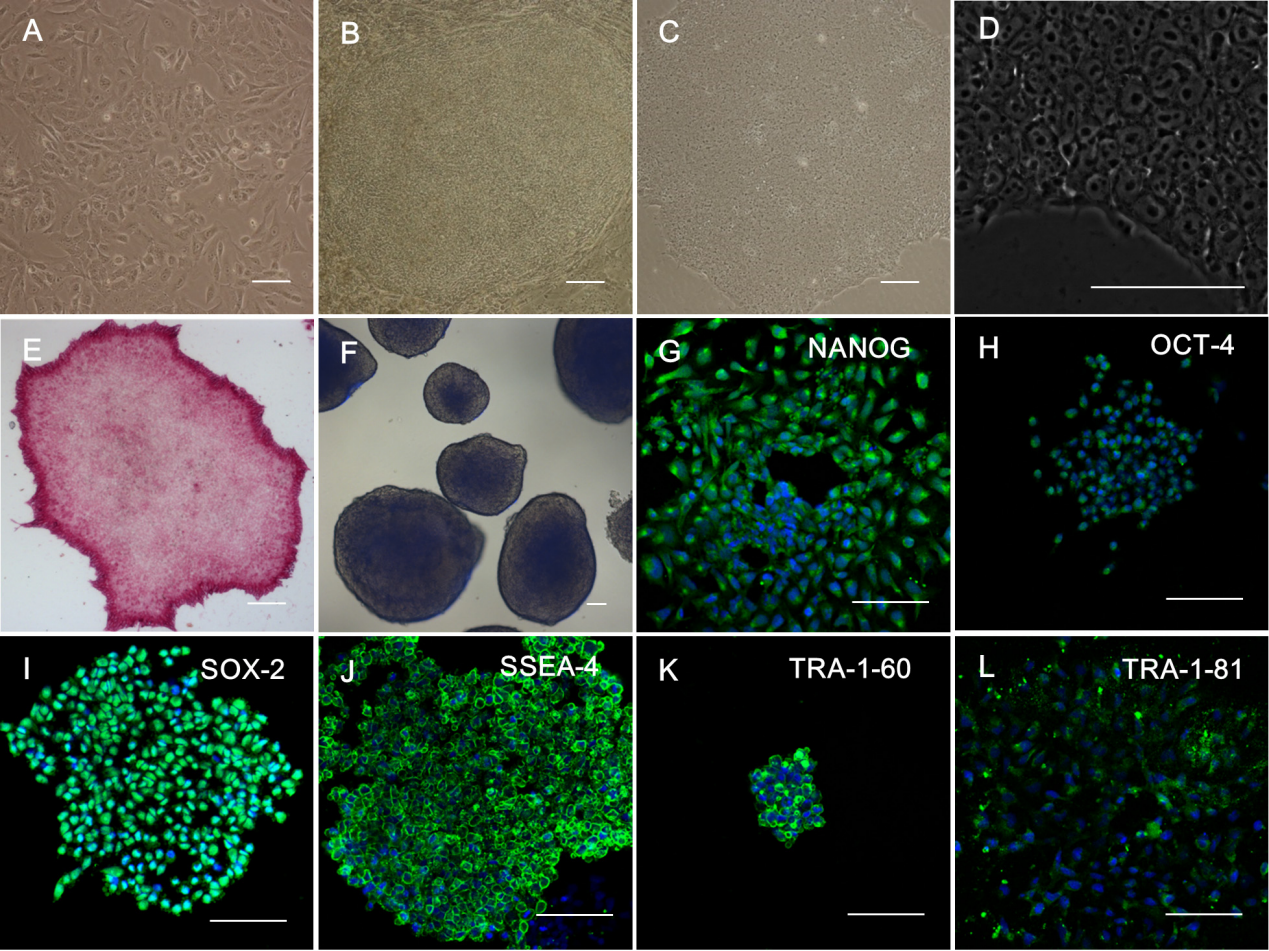
Induction of iPSCs from SFMSCs (Patient A). (A) Microscopic image showing the morphology of SFMSCs after transfection for 4 days. (B) Microscopic image showing the morphology of SFMSCs after transfection for 20 days. (C) Typical image of hES cell-like colony after culture with Matrigel and mTeSR1 medium culture. (D) High-magnification image of SFMSC-iPSCs. (E) Staining of SFMSC-iPSCs showing positive expression of alkaline phosphatase. (F) Floating culture of SFMSC-iPSCs at day 8. Immunofluorescent staining of SFMSC-iPSCs showing positive expression of NANOG (G), OCT-4 (H), SOX-2 (I), SSEA-4 (J), TRA-1-60 (K), and TRA-1-81 (L). Scale bars = 100 μm.

Four days after transfection, the mesenchymal-epithelial transformation (MET) was observed (Fig 3A). Twenty days after transfection, typical hES cell-like colonies were observed in culture (Fig 3B). We then picked up hES cell-like cell clones and cultured cells in Matrigel-coated 6-well plates with mTeSR1 medium; we defined these cell colonies as SFMSC-iPSCs (passage number 1). The cell status of SFMSC-iPSCs was maintained well and stably in vitro (Fig 3C and 3D). AP staining showed that SFMSC-iPSCs exhibited alkaline phosphatase activity (Fig 3E, Figs A, I in S1 File). After seeding SFMSC-iPSCs in untreated 6-well plates with mTSeR1 medium for 8 days as a floating culture, EBs were formed (Fig 3F, Figs B, J in S1 File). According to immunofluorescent staining, SFMSC-iPSCs also expressed NANOG, OCT-4, SOX-2, SSEA-4, TRA-1-60, TRA-1-81, typical markers of hESs (Fig 3G–3L, Figs C–H, K–P in S1 File).

### Generation and characterization of SFMSC-iPSC-MSCs

**Fig 4:**
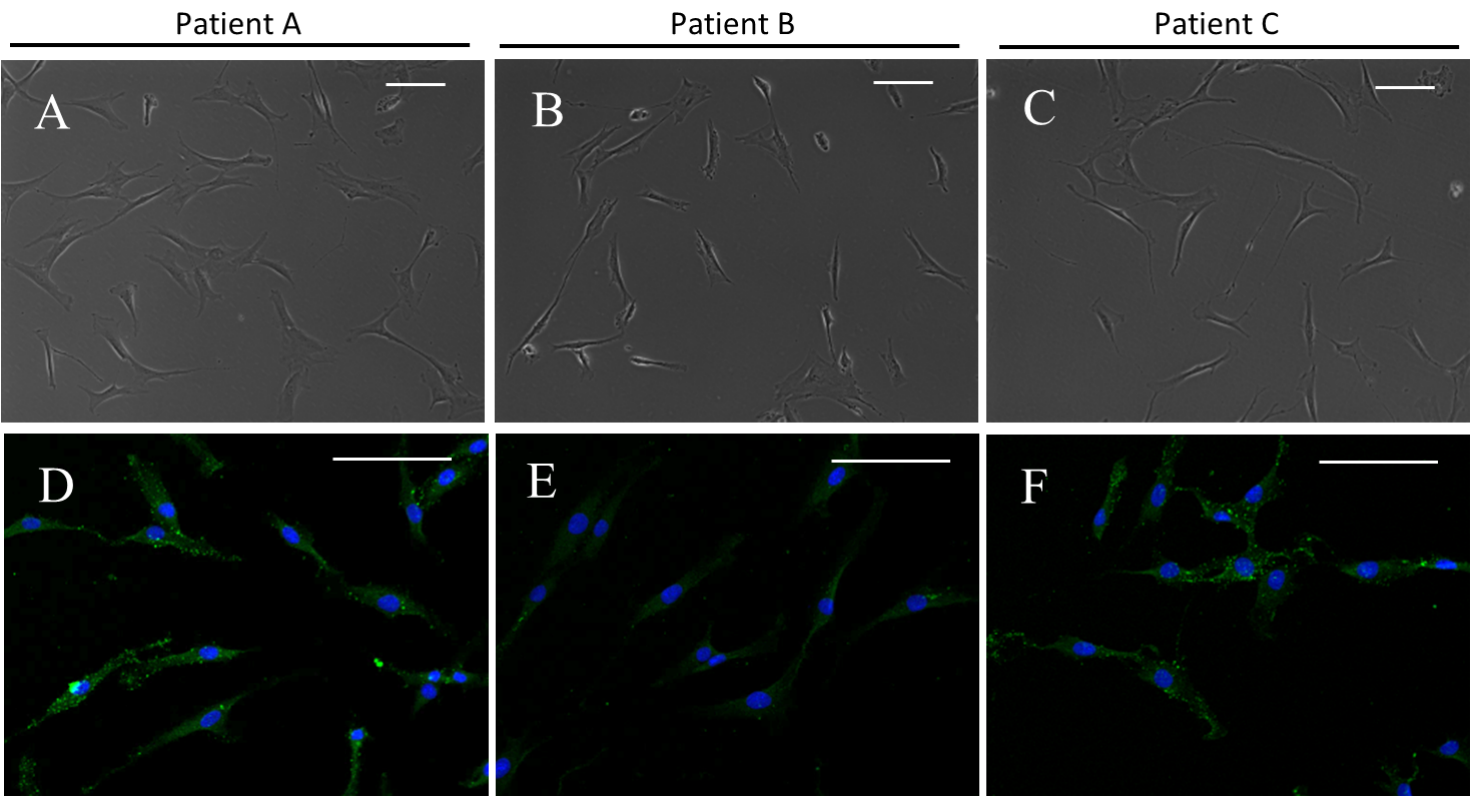
SFMSC-iPSC-MSCs. (A–C) Microscopic image showing the typical morphology of SFMSC-iPSC-MSCs. (D–F) Immunofluorescent staining of SFMSC-iPSC-MSCs showing positive expression of STRO-1 at passage 3. Scale bars = 100 μm.

After passaging in vitro, the cells exhibited a typical fibroblastic spindle shape (Fig 4A–4C). Flow cytometric analysis showed that SFMSC-iPSC-MSCs expressed typical surface markers of MSCs, such as CD90, CD105, CD73, and CD44. CD45, CD34, CD11b, CD19, and HLA-DR, were not detected (Fig 2). Interesting, STRO-1, which was not expressed in ancestor cells (SFMSCs) at passage 6, re-emerged on SFMSC-iPSC-MSCs (Fig 4D–4F).

### Cell proliferation potential of SFMSC-iPSC-MSCs and SFMSCs

**Fig 5:**
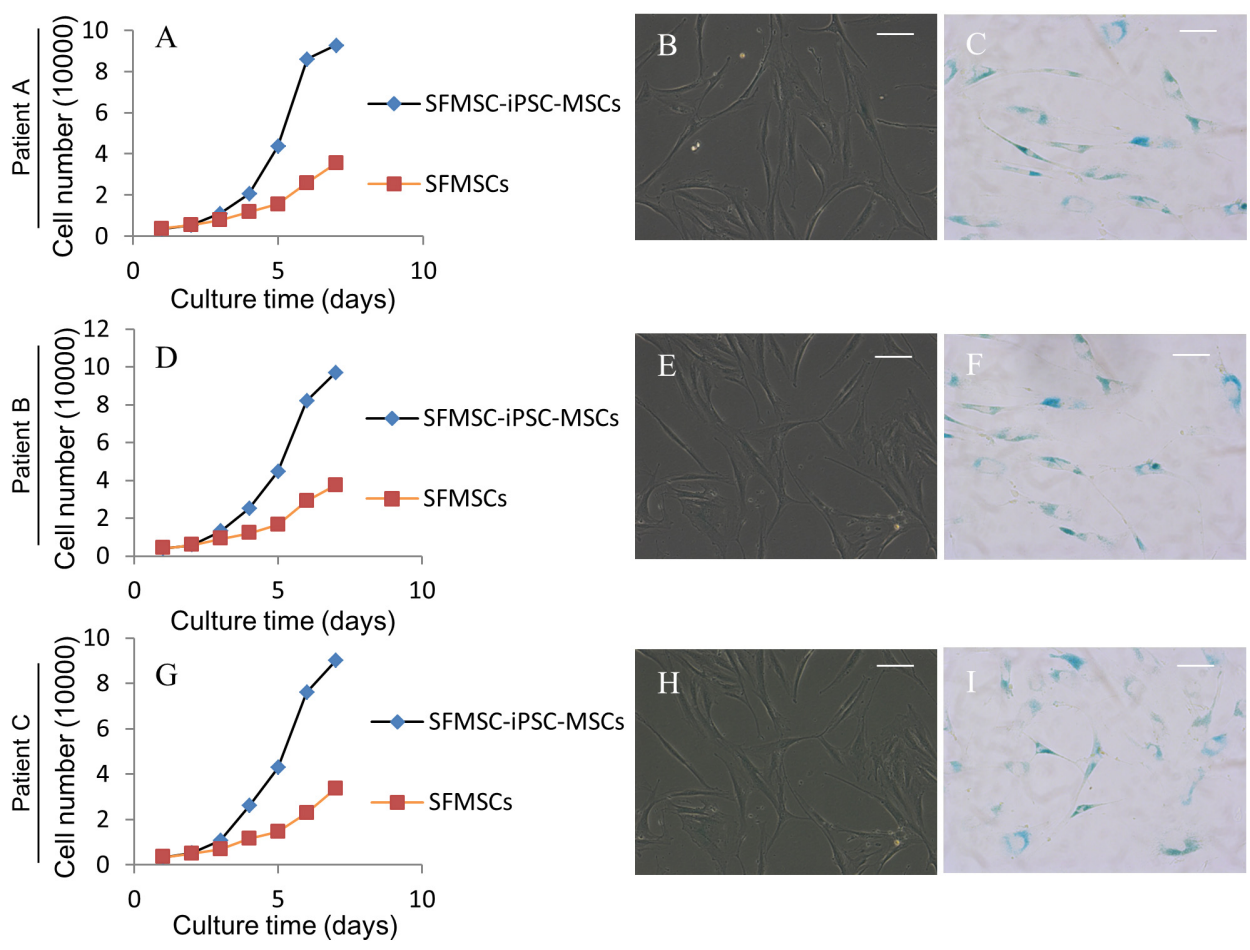
Cell growth curve and senescence assay of SFMSC-iPSC-MSCs and SFMSCs.

Cell growth curve showed that cell proliferation improved obviously after transformation (Fig 5A, 5D and 5G)). The average PD of SFMSCs (passage 6) was 1.72 ± 0.04, and SFMSC-iPSC-MSCs (passage 4) displayed an average PD of 2.81 ± 0.15. The average DTs were 41.85 ± 0.84 h for SFMSCs (passage 6) and 25.66 ± 1.40 h for SFMSC-iPSC-MSCs (passage 4). Accumulation of senescent cells was not obvious according to β-galactosidase staining in SFMSCs (passage 6). Accumulation of senescent cells was observed in SFMSC-iPSC-MSCs after passaging in vitro (Patient A, passage number 11 [Fig 5B and 5C]; Patient B, passage number 13 [Fig 5E and 5F]; Patient B, passage number 10 [Fig 5H and 5C]), indicating these cells were not immortalized.

### Differentiation of SFMSC-iPSC-MSCs and SFMSCs

#### Adipogenic differentiation

**Fig 6:**
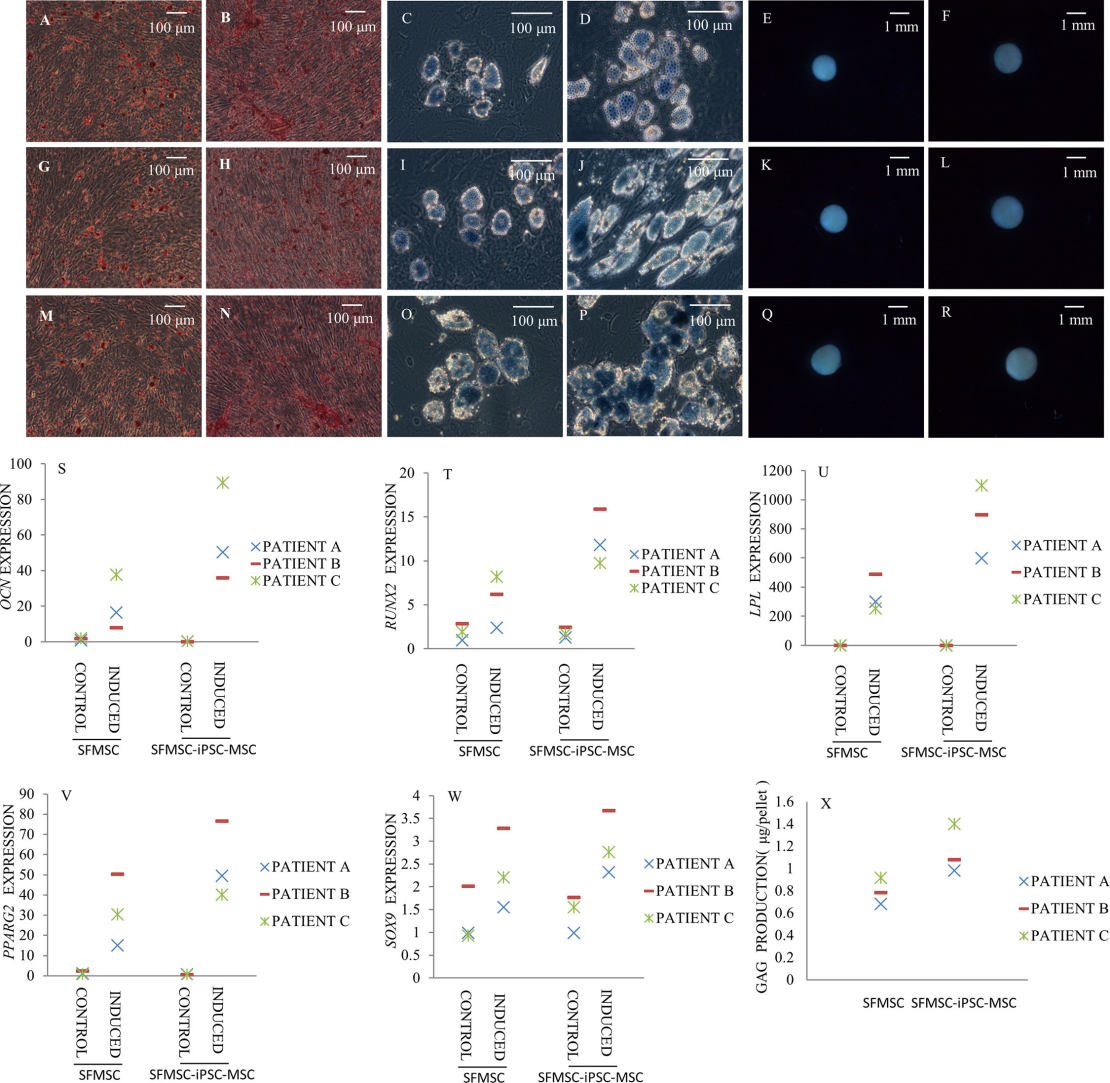
Multipotent differentiation of SFMSCs and SFMSC-iPSC-MSCs. Alizarin red staining identified calcium deposits in SFMSCs (A, G, M) and SFMSC-iPSC-MSCs (B, H, N) after osteogenic induction for 28 days. Lipid droplets were observed in SFMSCs (C, I, O) and SFMSC-iPSC-MSCs (D, J, P) after culture in adipogenic induction medium for 28 days by sudan black B staining. Cartilage nodules formed by SFMSCs (E, K, Q) and SFMSC-iPSC-MSCs (F, L, R) after chondrogenic induction for 21 days. *OCN* (S) and *RUNX-2* (T) gene expression in SFMSCs and SFMSC-iPSC-MSCs after osteogenic induction for 21 days. *LPL* (U) and *PPARG2* (V) gene expression in SFMSCs and SFMSC-iPSC-MSCs after adipogenic induction for 28 days. *SOX9* (W) gene expression and GAG (X) protein expression in SFMSCs and SFMSC-iPSC-MSCs after chondrogenic induction for 21 days.

After 28 days of culture in adipogenic induction medium, both SFMSCs and SFMSC-iPSC-MSCs developed into sudan black B-positive, lipid-laden fat cells (Fig 6C, 6D, 6I, 6J, 6O and 6P). Moreover, the expression levels of *LPL* and *PPARG2* genes in SFMSC-iPSC-MSCs were higher, respectively, than that in SFMSCs (Fig 6U and 6V).

#### Osteogenic differentiation

After 28 days of culture in osteogenic induction medium, calcium deposits were observed in both SFMSC and SFMSC-iPSC-MSC cultures by Alizarin Red staining (Fig 6A, 6B, 6G, 6H, 6M and 6N). Expression levels of the osteogenic transcription factor gene *RUNX-2* and the *OCN* gene in SFMSC-iPSC-MSCs were higher than that in SFMSCs, respectively, after osteogenic induction for 21 days (Fig 6S and 6T).

#### Chondrogenic differentiation

After 21 days of culture in chondrogenic induction medium, both SFMSCs and SFMSC-iPSC-MSCs formed cartilage nodules (Fig 6E and 6F); this effect was not observed in the respective control cell cultures. After 21 days of culture in chondrogenic induction medium, immunochemical staining for collagen type II was positive throughout sections of nodules formed by both cell types (Figs A-F in S3 File) whereas no immunostaining was detected in the respective control sections. Moreover, the expression levels of the *SOX9* gene and GAG protein in SFMSC-iPSC-MSCs were higher, respectively, than those in SFMSCs (Fig 6W and 6X).

### Karyotype analysis of SFMSC-iPSC-MSCs

**Fig 7:**
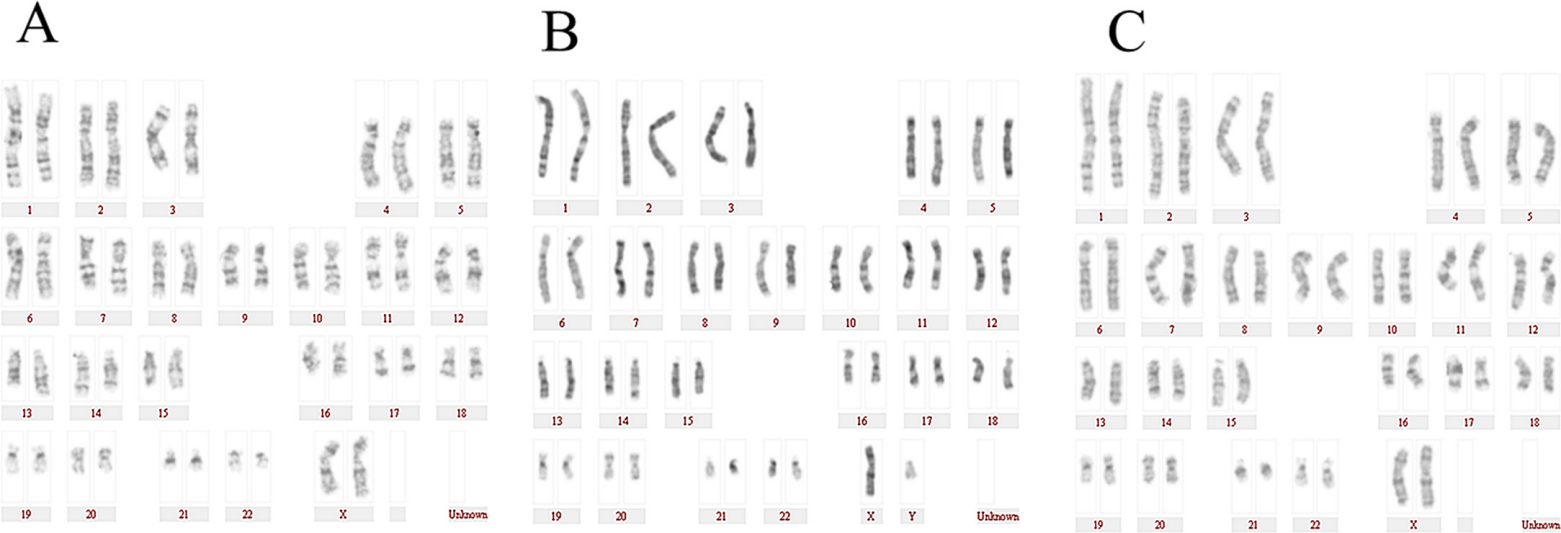
Karyotype analysis of SFMSC-iPSC-MSCs.

Y-27632 is essential for iPSC survival during single-cell dissociation. Single-cell dissociation with trypsin could induce genetic instability. However, in this study, the karyotype of SFMSC-iPSC-MSCs derived from patient A (Fig 7A), patient B (Fig 7B), patient C (Fig 7C) still remained normal.

### Tumor-generation assay *in vivo*

**Fig 8:**
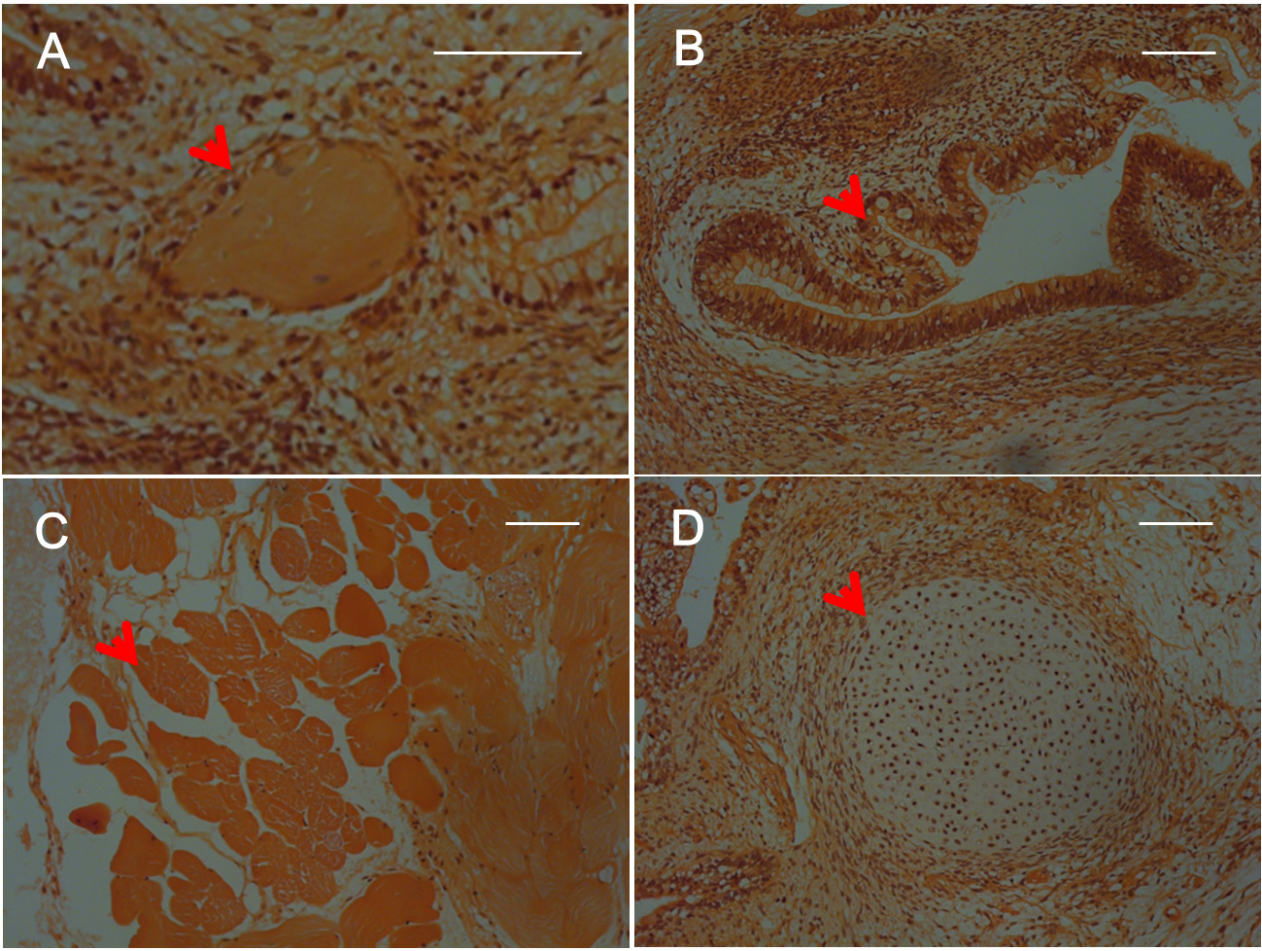
Hematoxylin and eosin staining of teratomas derived from SFMSC-iPSCs (Patient A). Scale bars = 100 μm.

At 12 weeks after implantation of SFMSC-iPSCs, tumor formation was observed in SCID mice. The tumors contained various tissues originating from three germ layers, including keratin-containing epidermal tissues (Fig 8A, Figs A, E in S2 File), gut-like epithelial tissues (Fig 8B, Figs B, F in S2 File), striated muscle (Fig 8C, Figs C, G in S2 File), and cartilage (Fig 8D, Figs D, H in S2 File), as shown by the results of histological examination. No side effects of injection of SFMSC-iPSC-MSCs were observed.

## Discussion

The local inflammatory milieu may affect the characteristics of MSCs [36]. Moreover, the inflammatory milieu of patients with TMD varies. In this study, we only chose three patients with TMD as donors of synovial fluid samples. Thus, this sample was very limited. Therefore, TMJ status and cytokine levels in patients with TMD who donated synovial fluid samples were assayed in this study.

In our preliminary study, the number of SFMSCs and the proliferation potential of the cells varied from patient to patient (data not shown). The cell number required for clinical approaches is very high, and this may be an obstacle for the clinical application of this technique. Therefore, for therapeutic application of these cells, identification of an alternative method for solving this dilemma is needed.

In this study, we reprogrammed SFMSCs that had lost STRO-1 and CD146 antigens during culture in vitro into iPSCs by reprogramming techniques with Sox2, Oct-3/4, klf4, and c-Myc. We then successfully transformed these iPSCs into MSCs on a Matrigel coating. These SFMSC-iPSC-MSCs exhibited improved proliferative activity and differentiation potential in vitro. These findings further support previous studies demonstrating that reprogrammed SFMSCs derived from iPSCs and controlled induction of iPSCs into MSCs may be an attractive approach to obtaining a readily available source of progenitor cells for therapeutic applications.

MSCs derived from different iPSC lines exhibit variability in their characteristics [27]. Thus, detailed characterization of iPSC-MSCs induced by specific methods is imperative. MSCs express a variety of surface antigens, including CD105 (an ecto-5′-nucleotidase originally recognized by monoclonal antibodies targeting SH3 and SH4), CD73 (an endoglin originally recognized by monoclonal antibodies targeting SH2), and CD90 (also known as Thy-1), and lack expression of CD34 (a primitive hematopoietic progenitor and endothelial cell marker), CD45 (a pan-leukocyte marker), CD79alpha/CD19 (a B cell marker), CD14/CD11b (prominently expressed on monocytes and macrophages), and HLA-DR (a marker only present in MSCs upon with stimulation with interferon-c), according to the minimal criteria proposed by The Mesenchymal and Tissue Stem Cell Committee of the International Society for Cellular Therapy (ISCT) for use as the uniform definition of human MSCs [37]. The MSC-like cells generated in this study met the ISCT’s criteria for defining an MSC population. STRO-1 is a surface marker for a purely primitive subset of MSCs and has not been confirmed as a definitive marker of MSCs due to the unstable and variably frequency of STRO-1 expression in MSCs. Indeed, STRO-1 expression is highly dependent on donor, culture conditions, and cell status [29]; however, MSCs that express STRO-1 have been shown to exert more powerful immunosuppressive effects in vitro and have greater homing ability in vivo [38,39]. Bakopoulou et al. [40] reported that STRO-1+/CD146+ stem cells residing in the apical papilla exhibited enhanced multipotent stem cell properties compared to the STRO-1-CD146+ stem cell subset. Koyama et al. [9] published the first report showing that STRO-1+CD146+SFMSCs, which had characteristics similar to those of bone marrow-derived MSCs, exist in the synovial fluid of the TMJ in patients with TMD. However, STRO-1 and CD146 antigen expression in SFMSCs disappeared easily following in vitro culture according to our previous studies [32]. In this study, we obtained SFMSCs from TMD patients; in these cells, STRO-1 was expressed in early passages, but disappeared quickly after a few passages in vitro. Furthermore, these SFMSCs showed poorer and poorer proliferative ability and multipotent differentiation potential during culture in vitro.

STRO-1+CD146+SFMSCs have been proposed to be an attractive autologous cell source for cell-based therapies and engineering strategies due to their extensive proliferative capacity, multipotent differentiation potential, and anti-inflammatory effects [9]. However, the unstable antigen expression, heterogeneity, and varying cell numbers of SFMSCs obtained from TMD patients significantly limit their therapeutic potential. Interestingly, with our protocol, we observed re-emergence of STRO-1 and CD146 expression.

Some recent studies have demonstrated the generation and characterization of MSCs from ESCs [16,19,22,23,25,41] or iPSCs [14,15,27]. Interestingly, the characteristics of MSCs derived from iPSCs or ESCs vary depending on the particular ancestor cells used or on the transformation protocol [27]. Chen et al. [15] demonstrated that iPSC-MSCs exhibited robust osteogenic and chondrogenic differentiation potential but limited adipogenic differentiation potential using the transforming growth factor-β pathway inhibitor SB431542. Lian et al. [42] obtained iPSC-MSCs successfully in a defined culture system using a combination of CD24 (-) and CD105 (+) sorting. The iPSC-MSCs exhibited highly efficient adipogenic, chondrogenic, and osteogenic differentiation potential. Olivier et al. [17] reported that MSCs obtained from ESCs in their study exhibited bipotent differentiation potential. CD146+STRO-1- MSCs could be derived from iPSCs based on fibrillar collagen coating culture and exhibited adipogenic, chondrogenic, and osteogenic differentiation potential [24]. Moreover, Wu et al. [19] reported that Nestin(+)/CD271(-)/STRO-1(-) mesenchymal-like precursors derived from hESCs in chemically defined conditions exhibited the ability to differentiate into osteoblasts, adipocytes, and chondrocytes.

In this study, we tried to reverse the status of SFMSCs that were CD146 negative and STRO-1 negative by reprogramming the SFMSCs into iPSCs by transduction with lentivirus-mediated Sox2, Oct-3/4, klf4, and c-Myc factors. These iPSCs showed characteristics similar to those of hESCs. These findings add further support to previous studies showing that SFMSCs could also be reprogrammed into iPSCs. Moreover, the SFMC-iPSC-MSCs obtained in our study exhibited typical characteristics of MSCs. Interestingly, CD146 and STRO-1 re-emerged on MSCs derived from SFMSC-iPSCs, and exhibit improved adipogenic, chondrogenic, and osteogenic differentiation potential, compared to their ancestor cells (SFMSCs). The molecular mechanisms underlying these characteristic changes remain unclear. A number of factors could have contributed to these characteristics of SFMSC-iPSC-MSCs. Notably, the iPSC lines used in this study were generated using combinations of transcription factors, including Sox2, Oct-3/4, klf4, and c-Myc. Moreover, we cannot exclude the role of reprogramming factors integrated in the genome. Further studies are required to confirm this using SFMSC-iPSCs derived from safer nonintegrative reprogramming systems. From our current work, we are unable to definitively show that the differences in these characteristic changes in SFMSC-iPSC-MSCs are a result of transformation of iPSCs into MSCs mediated by the Matrigel matrix.

The molecular mechanism mediating the transformation of iPSCs into MSCs remains poorly understood. To induce iPSC differentiation to MSCs, dissociated SFMSC-iPSCs were treated with the ROCK inhibitor Y-27632, which has been reported to decrease the dependence of hESCs on cell-cell contact and permit survival of single hESCs [43]. Therefore, we speculate that Y-27632 plays an important role in the transformation process. Therefore, Y-27632 may offer more opportunities for dissociated single SFMSC-iPSCs to interact with Matrigel matrix, thereby enhancing the effects of Matrigel matrix on the differentiation process by decreasing the apoptosis rates of dissociated single SFMSC-iPSCs.

The major components of the Matrigel matrix are entactin, collagen IV, laminin, and heparan sulfate proteoglycan. The matrix also contains plasminogen activators, growth factors, and other undefined components. The role of Matrigel in this transformation process remains unclear. However, the cell statuses of dissociated single SFMSC-iPSCs plated in plates coated with Matrigel matrix were better than those plated in plates without any matrix coating (data not show). Because biomaterials can influence cell fate through physicochemical stimulation [44,45], we hypothesize that Matrigel matrix may promote the transformation of SFMSC-iPSCs into MSCs through physicochemical stimulation to SFMSC-iPSCs. Further studies are needed to elucidate the details of these mechanisms.

## Conclusion

This study further supports previous studies showing SFMSCs can also be reprogrammed into iPSCs by transduction with lentivirus-mediated Sox2, Oct-3/4, klf4, and c-Myc factors. STRO-1 and CD146 expression on SFMSCs was highly dependent on the patient and culture condition and disappeared easily during in vitro culture. Thus, we found that uniform, dynamic MSCs could be obtained from SFMSC-iPSCs on plates coated with Matrigel matrix. The generated SFMSC-iPSC-MSCs showed typical MSC characteristics and more dynamic characteristics than their ancestor cells. Moreover, STRO-1 and CD146 expression re-emerged on SFMSC-iPSC-MSCs. These data provide important insights into optimal culture conditions for obtaining MSCs from SFMSC-iPSCs.

## Supporting Information

S1 File Induction of iPSCs from SFMSCs (Patients B and C). Staining of SFMSC-iPSCs showing positive expression of alkaline phosphatase (Fig A, Fig I). Floating culture of SFMSC-iPSCs at day 8 (Fig B, Fig J). Immunofluorescent staining of SFMSC-iPSCs showing positive expression of NANOG (Fig C, Fig K), OCT-4 (Fig D, Fig L), SOX-2 (Fig E, Fig M), SSEA-4 (Fig F, Fig N), TRA-1-60 (Fig G, Fig O), and TRA-1-81 (Fig H, Fig P). Scale bars = 100 μm.
(TIF)

S2 File Hematoxylin and eosin staining of teratomas derived from SFMSC-iPSCs (Patients B and C). Scale bars = 100 μm.
(TIF)

S3 File Collagen type II immunohistochemical staining of cartilage nodules. Cartilage nodules formed by SFMSCs (Fig A, Fig B, Fig C) and SFMSC-iPSC-MSCs (Fig D, Fig E, Fig F) after induction for 3 weeks. Scale bars = 100 μm.
(TIF)
