# Supplementary figures and images for: Mesenchymal Stem Cells Obtained from Synovial Fluid Mesenchymal Stem Cell-Derived Induced Pluripotent Stem Cells on a Matrigel Coating Exhibited Enhanced Proliferation and Differentiation Potential
Source: PLoS One. 2015 Dec 9;10(12):e0144226. doi: 10.1371/journal.pone.0144226 (PMC4674106; doi:10.1371/journal.pone.0144226)

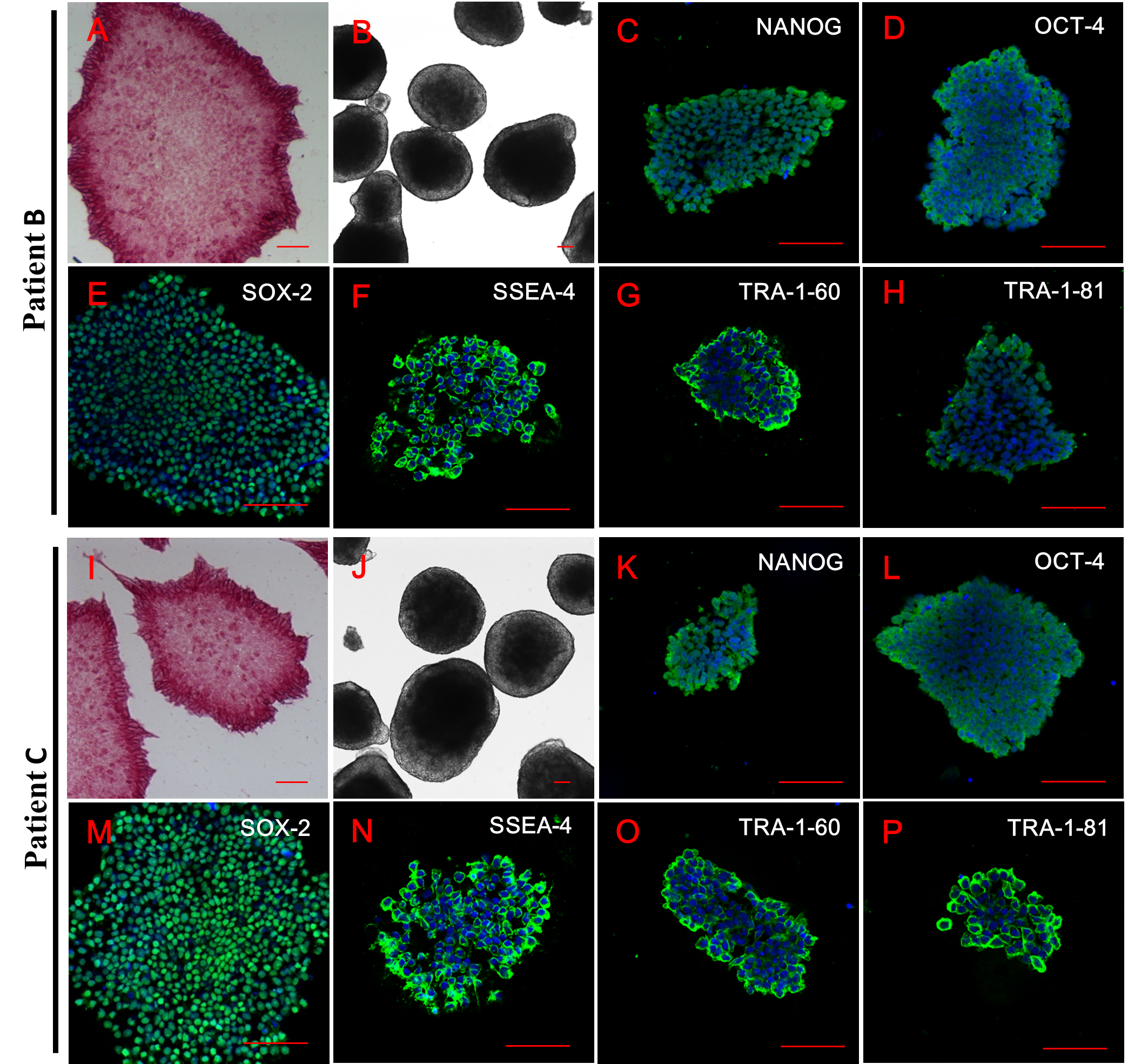

Supplement: S1 File — Staining of SFMSC-iPSCs showing positive expression of alkaline phosphatase (Fig A, Fig I). Floating culture of SFMSC-iPSCs at day 8 (Fig B, Fig J). Immunofluorescent staining of SFMSC-iPSCs showing positive expression of NANOG (Fig C, Fig K), OCT-4 (Fig D, Fig L), SOX-2 (Fig E, Fig M), SSEA-4 (Fig F, Fig N), TRA-1-60 (Fig G, Fig O), and TRA-1-81 (Fig H, Fig P). Scale bars = 100 μm. (TIF) [file pone.0144226.s001.tif]

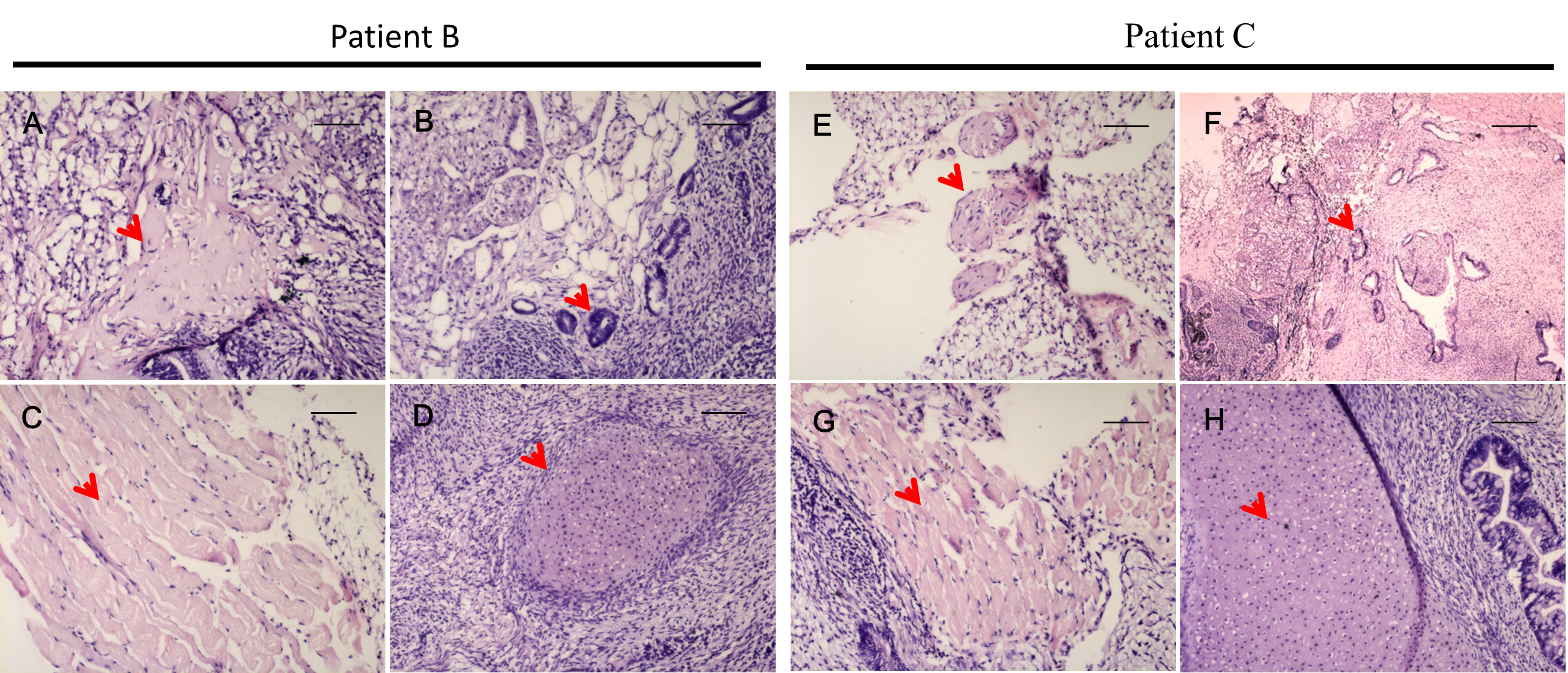

Supplement: S2 File — Scale bars = 100 μm. (TIF) [file pone.0144226.s002.tif]

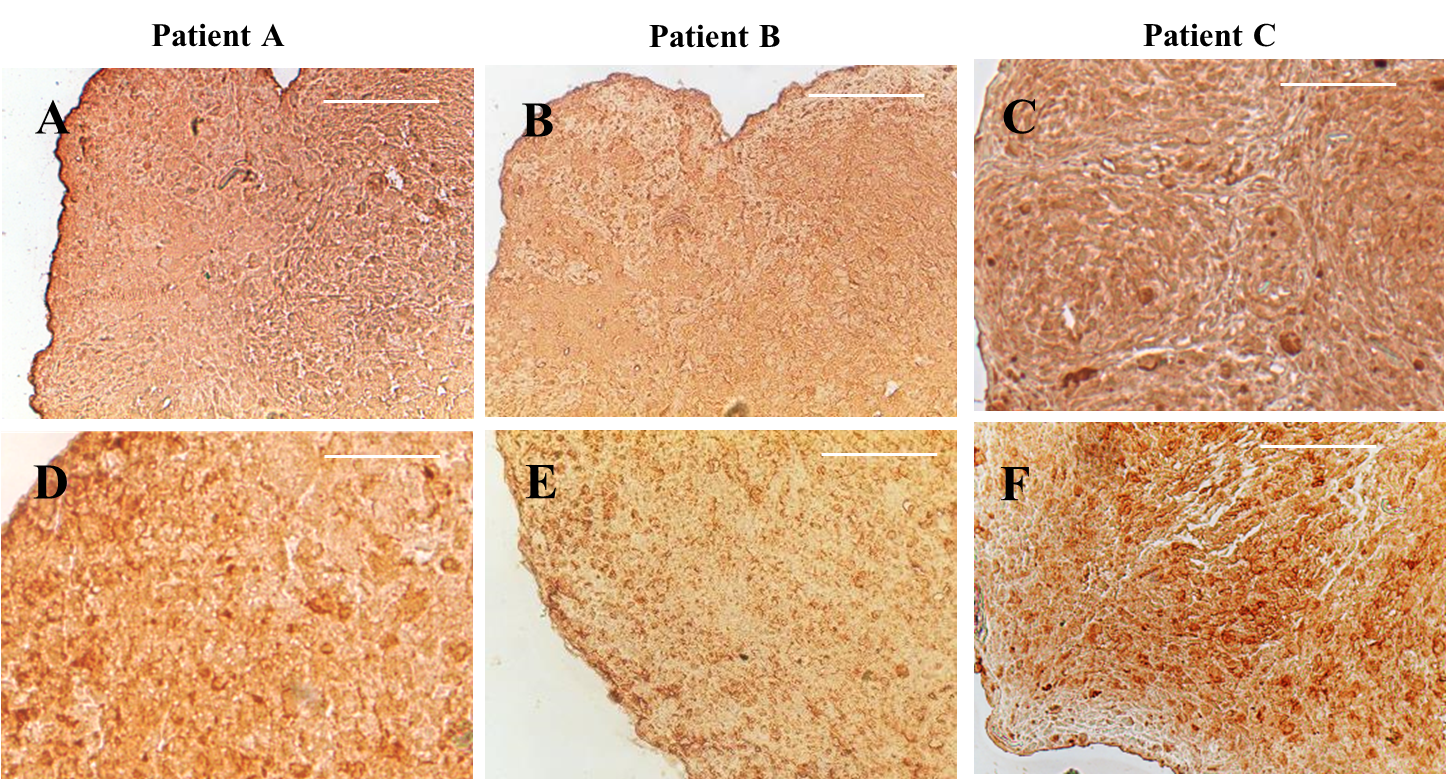

Supplement: S3 File — Cartilage nodules formed by SFMSCs (Fig A, Fig B, Fig C) and SFMSC-iPSC-MSCs (Fig D, Fig E, Fig F) after induction for 3 weeks. Scale bars = 100 μm. (TIF) [file pone.0144226.s003.tif]
